# Ultrastructural Assessment and Proteomic Analysis in Myofibrillogenesis in the Heart Primordium After Heartbeat Initiation in Rats

**DOI:** 10.3389/fphys.2022.907924

**Published:** 2022-05-09

**Authors:** Nobutoshi Ichise, Tatsuya Sato, Hiroyori Fusagawa, Hiroya Yamazaki, Taiki Kudo, Izaya Ogon, Noritsugu Tohse

**Affiliations:** ^1^ Department of Cellular Physiology and Signal Transduction, Sapporo Medical University School of Medicine, Sapporo, Japan; ^2^ Department of Cardiovascular, Renal, and Metabolic Medicine, Sapporo Medical University School of Medicine, Sapporo, Japan; ^3^ Department of Orthopaedic Surgery, Sapporo Medical University School of Medicine, Sapporo, Japan

**Keywords:** myofibril assembly, sarcomere formation, excitation-contraction coupling, data-independent acquisition mass spectrometry, heart primordium, primitive heart tube

## Abstract

Myofibrillogenesis is an essential process for cardiogenesis and is closely related to excitation-contraction coupling and the maintenance of heartbeat. It remains unclear whether the formation of myofibrils and sarcomeres is associated with heartbeat initiation in the early embryonic heart development. Here, we investigated the association between the ultrastructure of myofibrils assessed by transmission electron microscopy and their proteomic profiling assessed by data-independent acquisition mass spectrometry (DIA-MS) in the rat heart primordia before and after heartbeat initiation at embryonic day 10.0, when heartbeat begins in rats, and in the primitive heart tube at embryonic day 11.0. Bundles of myofilaments were scattered in a few cells of the heart primordium after heartbeat initiation, whereas there were no typical sarcomeres in the heart primordia both before and after heartbeat initiation. Sarcomeres with Z-lines were identified in cells of the primitive heart tube, though myofilaments were not aligned. DIA-MS proteome analysis revealed that only 43 proteins were significantly upregulated by more than 2.0 fold among a total of 7,762 detected proteins in the heart primordium after heartbeat initiation compared with that before heartbeat initiation. Indeed, of those upregulated proteins, 12 (27.9%) were constituent proteins of myofibrils and 10 (23.3%) were proteins that were accessories and regulators for myofibrillogenesis, suggesting that upregulated proteins that are associated with heartbeat initiation were enriched in myofibrillogenesis. Collectively, our results suggest that the establishment of heartbeat is induced by development of bundles of myofilaments with upregulated proteins associated with myofibrillogensis, whereas sarcomeres are not required for the initial heartbeat.

## Introduction

The embryonic heart functions ahead of all other organs to support whole embryonic development by maintaining fetal circulation, and thus “the initiation of heartbeat” is a critical event in the light of the birth of life. However, compared to the well-studied morphological features of heart development, the underlying mechanism by which the first heartbeat begins remains unclear. Although it has been reported that the timeline of embryonic development (Hill. 2022) and the timing of the initial heartbeat vary among species (Hirota et al., 1985; Hirota et al., 1987; Nishii and Shibata. 2006; Tyser et al., 2021), our previous study showed that the heartbeat begins at embryonic day 9.99–10.13 (E9.99–10.13) in rats with a calcium transient *via* extracellular calcium influx preceding muscle contraction (Kobayashi et al., 2011). Our previous finding that a calcium transient occurred even before the heartbeat begins was supported by the results of another study (Tyser et al., 2016). For one of the possible mechanisms that contribute to the initiation of heartbeat, we recently demonstrated that enhanced glycolysis and glucose oxidation *via* activation of hypoxia-inducible factor 1α (HIF-1α) cover the energy demand in a rat embryonic heart primordium after heartbeat initiation (Sato et al., 2022). However, the exact mechanism by which calcium transients occur without contractile activity in the embryonic heart is still unknown.

Myofibrils are fundamental components of striated muscle and are composed of proteins such as actin, myosin, and titin as well as proteins that bind them together. In matured striated muscle such as cardiac muscle, myofilaments are regularly arranged to form a functional contractile apparatus called a sarcomere, which is responsible for powerful contractile activity. Indeed, mutation of genes encoding component proteins of myofibrils and sarcomeres has been established to be a major cause of cardiomyopathy and congenital heart disease (Van der Velden et al., 2019; Martin and Kirk. 2020; Bang et al., 2022). Thus, myofibrillogenesis including sarcomere formation in the developing heart are tightly regulated for normal cardiogenesis. Although it has been reported that the formation of myofibrils and sarcomeres occurs rapidly in the period of the primitive heart tube, when heartbeat has already started (Nakajima et al., 2009; Tyser et al., 2021), the link between ultrastructural characteristics and molecular expression in myofibrillogenesis of the embryonic heart primordium in the period around the initial heartbeat has not been elucidated.

Recently, proteomics technology has dramatically improved detection sensitivity and quantitative reproducibility with the development of mass spectrometry (MS). Compared to conventional proteomic analysis such as the data-dependent acquisition (DDA) method, the data-independent acquisition (DIA) method acquires all MS/MS spectra regardless of protein expression levels and matches them with a pre-built spectral library, enabling more sensitive, reproducible, and accurate proteome analysis (Amodei et al., 2019; Kawashima et al., 2019). Indeed, little is known about protein expression patterns compared with recent advances in gene expression patterns during early embryonic organogenesis including the heart (Tyser et al., 2021; Lohoff et al., 2022; Sato et al., 2022). Therefore, a proteomics approach using the DIA method may enable comprehensive evaluation of trace changes in the expression of proteins such as component proteins of myofibrils and sarcomeres in the heart primordium around the timing of the initial heartbeat.

In the present study, we assessed morphological characteristics of myofibrils and sarcomeres by transmission electron microscopy in the rat heart primordia before and after heartbeat initiation at E10.0 and in the primitive heart tube at E11.0, and we comprehensively analyzed protein expression involved in these components using the DIA-MS method. Our results provide novel insights into the relationship between morphological features and protein expression patterns of myofibrils and sarcomeres in early cardiac development during the phase of heartbeat initiation in rats.

## Methods

### Animal Preparation

All animal studies were approved by the Committee for Animal Research, Sapporo Medical University (19–010). Wistar rats were purchased from Sankyo Labo Service (Sapporo, Japan) and were kept in the temperature-controlled animal facility at Sapporo Medical University. Since our previous study (Kobayashi et al., 2010) showed that the initial heartbeat in a rat embryo begins at E9.99–10.13 embryonic day (E9.99–10.13), which corresponds to midnight in a normal light-dark cycle, rats were housed in a light-dark reversal room as previously described (Sato et al., 2022). For mating, one female and two male rats were placed in the same breeding cage and successful mating was identified by the presence of a vaginal plug in the next light period. Embryonic day 0 (E 0) was defined as the midpoint of the previous dark period. A pregnant rat carrying embryos at E10.0 or E11.0 was sacrificed by carbon dioxide euthanasia, and the uterus with embryos was quickly removed and was placed in a small incubator (MI-IBC, Olympus, Tokyo, Japan) containing phosphate buffered saline (PBS) at room temperature for transmission electron microscopy (TEM) or on ice for proteomics experiments. Embryos were isolated from the uterus and their heart primordia at E10.0 or primitive heart tubes at E11.0 were dissected under a microscope in the incubator. Samples of heart primordia or primitive heart tubes were used for the subsequent procedure for TEM or were freshly frozen in liquid nitrogen and stored at −80°C until the assay.

### Definition of the Heart Primordium Before or After Heartbeat Initiation

**FIGURE 1:**
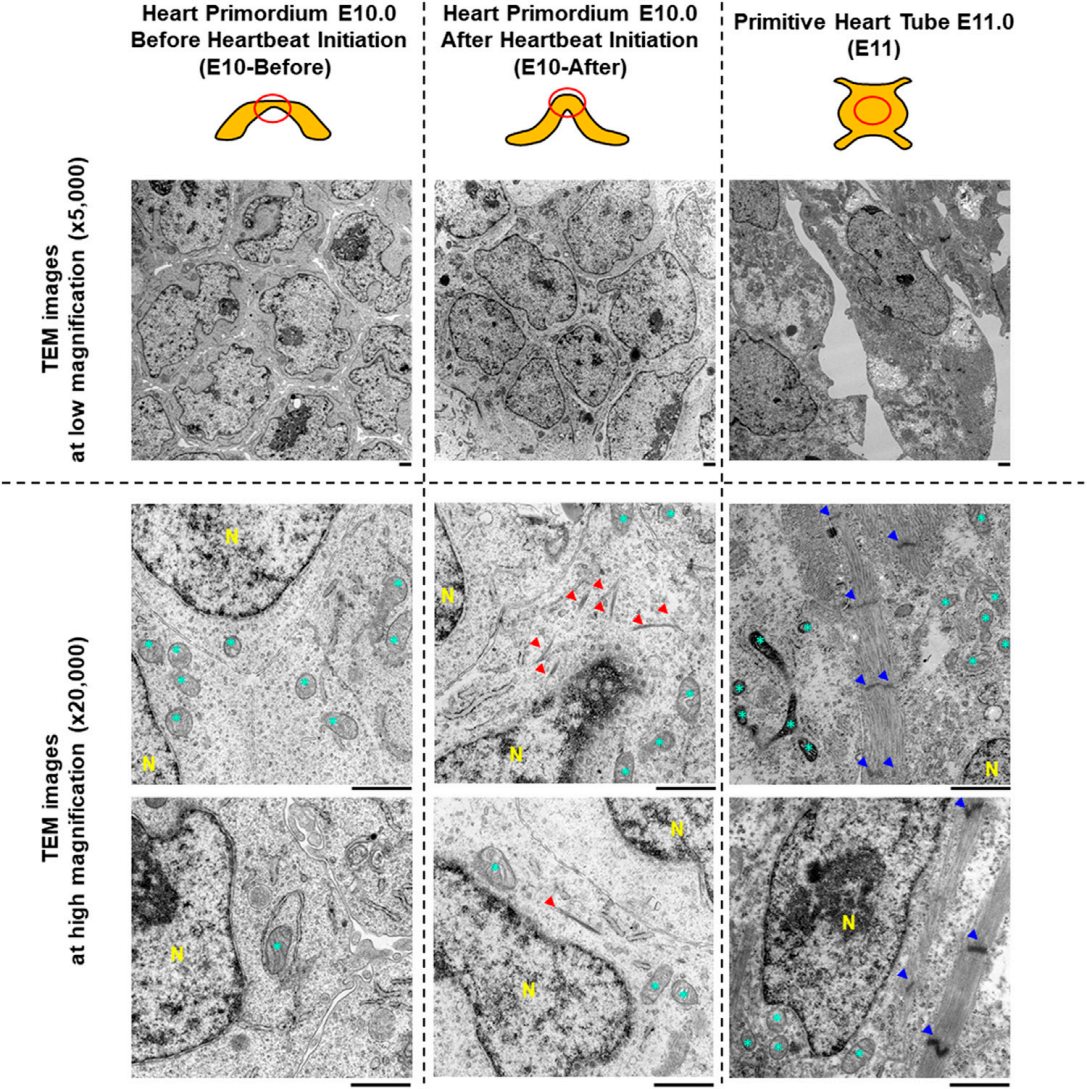
Characteristics of myofibrils and sarcomeres assessed by transmission electron microscopy in the heart primordium before and after heartbeat initiation and in the primitive heart tube. The upper illustrations represent the graphical shapes of the heart primordium at E10.0 before and after heartbeat initiation and the primitive heart tube at E11.0. The heart primordium before heartbeat initiation was defined as a heart primordium with a flat center shape and the heart primordium after heartbeat initiation was defined as a heart primordium with a convex and thickened shape. The red circle indicates the region of TEM observation. Representative TEM images at low magnification (×5,000, upper images) and at high magnification (×25,000, bottom images) are shown. Red arrowheads indicate bundles of myofilaments. Blue arrowheads indicate a Z-line of the sarcomere. * = mitochondria. N = nucleus. Scale bars under the images indicate 1.0 μm.

In accordance with the previous reports (Nakajima et al., 2009; Kobayashi et al., 2011; Tyser et al., 2021; Sato et al., 2022), embryos at E10.0 with a heart primordium showing a flat center were defined as embryos before heartbeat initiation, and those showing a convex thickened center were defined as embryos after heartbeat initiation in the present study (Figure 1). To minimize the potential for proteolysis of the heart primordium samples for proteomics experiments, embryos were immediately placed in ice-cold PBS and the presence or absence of beating was thus not examined to distinguish the heart primordium before and after heartbeat initiation in the present study.

### Transmission Electron Microscopy

The procedure used for TEM was previously described (Sato et al., 2022). In brief, the heart primordium at E10.0 or the primitive heart tube at E11.0 was fixed in 2.5% glutaraldehyde and 0.1 M sodium cacodylate at 4°C overnight, followed by treatment with 1% osmium tetroxide in 0.1 M sodium cacodylate. After dehydration, the samples were embedded in the mold of an epoxy resin block and cut with a microtome. The samples were finally stained with uranyl acetate and lead citrate and were examined by an electron microscope (H7650, Hitachi, Japan).

### Data-Independent Acquisition Proteomics Protein lysate Preparation

Four pooled embryonic heart primordia at E10.0 or primitive heart tubes at E11.0 were homogenized with CelLytic™ MT Cell Lysis Reagent (Sigma-Aldrich, St. Louis, MO) with proteinase inhibitor cocktail (Promega, Madison, WI) and the lysate was centrifuged at 3,000 g for 10 min at 4°C to obtain the supernatant as one sample. The protein concentration of the lysate was measured by a BCA assay (Takara-bio, Japan) and was adjusted to 0.5 μg/μl. The protein lysates were frozen on dry-ice and were shipped to Kazusa DNA Research Institute. Three samples each in the heart primordium before heartbeat initiation, the heart primordium after heartbeat initiation, and primitive heart tube were provided for proteomics.

### Sample Preparation for Proteomic Analysis

Sample preparation was performed as previously described with slight modifications (Nakamura et al., 2021). The sample was precipitated in 4-fold acetone and incubated at −20°C for 2 h. After centrifugation at 15,000 g for 15 min at 4°C, the pellet was extracted in 100 mM Tris-HCL pH 8.5, 2% SDS, pH 8.5 by using a water bath-type sonicator (Bioruptor II, CosmoBio, Tokyo, Japan) on the high setting for 15 min. The extracted proteins were measured by using a BCA protein assay kit (Thermo Fisher Scientific, Waltham, MA) and adjusted to 1.0 μg/μl with 100 mM Tris-HCL pH 8.5, 2% SDS, pH 8.5. To cleave the S-S bond of the protein, 20 μg protein extract was treated with 20 mM Tris (2-carboxyethyl) phosphine hydrochloride (TCEP) at 80°C for 10 min. The sample was then subjected to alkylation with 30 mM iodoacetamide in the dark at room temperature for 30 min and subjected to clean-up and digestion with single-pot solid phase-enhanced sample preparation. Briefly, two types of beads (hydrophilic and hydrophobic Sera-Mag Speed-Beads; Cytiva, Marlborough, MA, United States) were used. These beads were combined at a 1:1 (v/v) ratio, rinsed with distilled water, and reconstituted in distilled water at 15-μg solids/μl. The reconstituted beads (20-μl) were then added to the alkylated sample followed by ethanol to bring the final concentration to 75% (v/v), with mixing for 5 min. The supernatant was discarded, and the pellet was rinsed twice with 80% ethanol. The beads were then resuspended in 100 μl 50 mM Tris-HCl pH 8.0 with 1.0 µg trypsin and Lys-C Mix (Promega, Madison, WI, United States) and digested by gentle mixing at 37°C overnight. The digested sample was acidified with 20 μl 5% trifluoroacetic acid (TFA) and then sonicated with Bioruptor II (CosmoBio) at a high level for 5 min at room temperature. The sample was desalted using SDB-STAGE tip (GL Sciences Inc, Tokyo, Japan) according to the manufacturer’s instructions, followed by drying with a centrifugal evaporator. The dried peptides were redissolved in 2.0% acetonitrile and 0.1% TFA. The redissolved sample was adjusted for a peptide concentration of 150 ng/μl using a Lunatic instrument (Unchained Labs, Pleasanton, CA, United States) and transferred to a hydrophilic-coated, low-adsorption vial (ProteoSave vial; AMR Inc, Tokyo, Japan).

### Data-Independent Acquisition Mass Spectrometry

Peptides were directly injected onto a 75 μm × 12 cm nanoLC nano-capillary column (Nikkyo Technos Co., Ltd, Tokyo, Japan) at 40°C and then separated with an 80-min gradient at a flow rate of 200 nl/min using an UltiMate 3,000 RSLCnano LC system (Thermo Fisher Scientific, Waltham, MA). Peptides eluting from the column were analyzed on a Q Exactive HF-X (Thermo Fisher Scientific) for overlapping window DIA-MS (Amodei et al., 2019; Kawashima et al., 2019). MS1 spectra were collected in the range of 495–785 m/z at 30,000 resolution to set an automatic gain control target of 3 × 10^6^ and maximum injection time of 55. MS2 spectra were collected in the range of more than 200 m/z at 15,000 resolution to set an automatic gain control target of 3 × 10^6^, maximum injection time of “auto”, and stepped normalized collision energies of 22, 26, and 30%. The isolation width for MS2 was set to 4 m/z and overlapping window patterns in 500–780 m/z were used as window placements optimized by Skyline v20.2.0.34 (MacLean et al., 2010). Technical replicates were not performed in this experiment.

### Data Analysis

The MS files were searched against rat spectral libraries using Scaffold DIA (Proteome Software, Inc, Portland, OR, United States). Spectral libraries were generated from the rat protein sequence database (UniProt id UP000002494, 21589 entries, downloaded on 26 November 2021) by Prosit (Gessulat et al., 2019; Searle et al., 2020). Scaffold DIA search parameters were as follows: experimental data search enzyme, trypsin; maximum missed cleavage sites, one; precursor mass tolerance, 10-ppm; fragment mass tolerance, 10-ppm; static modification, and cysteine carbamidomethylation. The threshold for protein identification was set so that both protein and peptide false discovery rates (FDRs) were less than 1%. The protein and peptide quantification values were calculated using the Scaffold DIA. The protein quantification data were log2 transformed (protein intensities) and filtered so that for each protein, at least one group contained a minimum of 70% valid values. The remaining missing values were imputed by random numbers drawn from a normal distribution (width, 0.3; downshift, 1.8) in Perseus v1.6.15.0 (Tyanova et al., 2016). The thresholds for altered proteins were a more than 2.0-fold change and *p* < 0.05 (Welch’s *t*-test) that differed between the two groups.

## Results

### Bundles of Myofilaments are Identified in Cells of the Heart Primordium After Heartbeat Initiation but not in Cells Before Heartbeat Initiation

To assess myofibrillogenesis and sarcomere formation in cells of the heart primordia before and after heartbeat initiation, we observed cells in the heart primordium of embryos at E10.0 using TEM. As shown in Figure 1, there were no myofilaments or myofibril-like structures in 81 observed cells of the four heart primordia before heartbeat initiation, whereas bundles of myofilaments were identified in four of 134 observed cells of the three heart primordia after heartbeat initiation. Sarcomere structures defined by Z-lines were not observed in cells of the heart primordium before and after heartbeat initiation. Since we did not find sarcomeres or sarcomere-like structures in cells of the heart primordium of embryos at E10.0, we extended our TEM observations to cells of the primitive heart tube of embryos at E11.0. We found abundant myofibrils with Z-lines in 27 of 37 observed cells of the three primitive heart tubes of embryos at E11.0, but the myofilaments were not well-aligned and M-bands, which should be located in the middle of the sarcomere, were obscured. These results suggest that the development of bundles of myofilaments is associated with heartbeat initiation in the heart primordium, but sarcomere formation, which was identified at the developmental phase of the primitive heart tube, is not necessary for the initiation of heartbeat.

### Upregulated Proteins Associated With Myofibrillogenesis Are Highly Enriched in the Heart Primordium After Heartbeat Initiation Compared With Those Before Heartbeat Initiation

**FIGURE 2:**
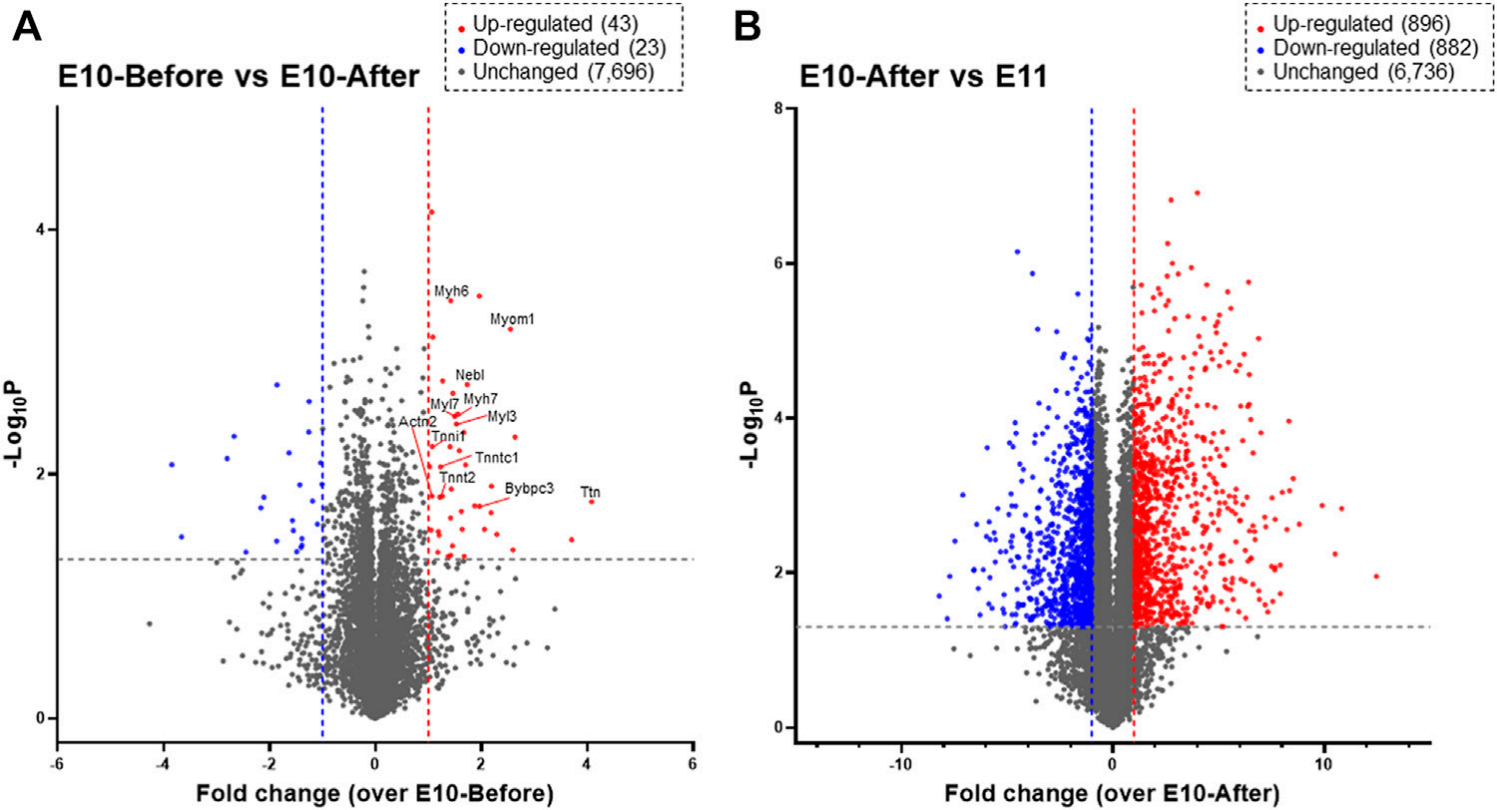
Proteomic profiling assessed by DIA-MS related to initial heartbeat in the heart primordium and to the development from the heart primordium to the primitive heart tube. Proteins that are expressed in the heart primordium before and after heartbeat initiation **(A)** and those expressed in the heart primordium after heartbeat initiation and the primitive heart tube **(B)** are shown by a volcano plot. The volcano plot represents the relationship between the magnitude (*X*-axis) and statistical significance (*Y*-axis) of altered protein expression. Colored plots represent expressed proteins with both a magnitude of change of more than 2.0 fold (Red: upregulated, Blue: downregulated) and significant change (*p* value < 0.05).

Next, we performed DIA-MS proteome analysis for the heart primordium at E10.0 to elucidate protein profiling in myofibrillogenesis that is associated with heartbeat initiation in the heart primordium. As shown in Figure 2A, proteins for which both a peptide false discovery rate (FDR) < 1% and a protein FDR <1% were satisfied were identified. Among a total of 7,762 recruited proteins in the heart primordium at E10.0, only 43 proteins (0.55%) were significantly upregulated by more than 2.0 fold and 23 proteins (0.30%) were significantly downregulated by more than 2.0 fold in the heart primordium after heartbeat initiation compared with those in the heart primordium before heartbeat initiation. Interestingly, of those 43 upregulated proteins, 12 proteins (27.9%) were constituent proteins of myofibrils including Myl3, Myl7, Myh6, Myh7, Actn2, Tnnt2, Tnni1, Tnnc1, Myom1, Mybpc3, Nebl, and Ttn. In addition, 10 of the 43 upregulated proteins (20.9%) were proteins that were accessories and regulators for myocardial contractile apparatuses including Smyd1 (Tan et al., 2006.), Synpo2l (Clausen et al., 2021), Mtpn (Sen et al., 1990), Csrp3 (Walsh et al., 2022), Unc45b (Myhre et al., 2014), Sox5 (Smits et al., 2001), Abra (Wallace et al., 2012), Mylk3 (Seguchi et al., 2007), Hspb2 (Sugiyama et al., 2000), and Lmod1 (Nworu et al., 2015). These findings indicate that proteins with increased expression that are associated with myofibrillogenesis are highly enriched in the heart primordium after heartbeat initiation compared with those in the heart primordium before heartbeat initiation. In contrast to the comparison between before and after heartbeat initiation in the heart primordium, 896 proteins (11.3%) were upregulated and 882 proteins (11.1%) were downregulated among 7,914 detected proteins when comparing proteins in the already-beating heart primordium at E10.0 and those in the primitive heart tube, suggesting that the expression levels of numerous proteins are altered during differentiation and development from the already-beating heart primordium at E10.0 to the primitive heart tube at E11.0 (Figure 2B).

### Expression Patterns of Constituent Proteins of Myofibrils and Sarcomeres in the Heart Primordium and Those in the Primitive Heart Tube

**FIGURE 3:**
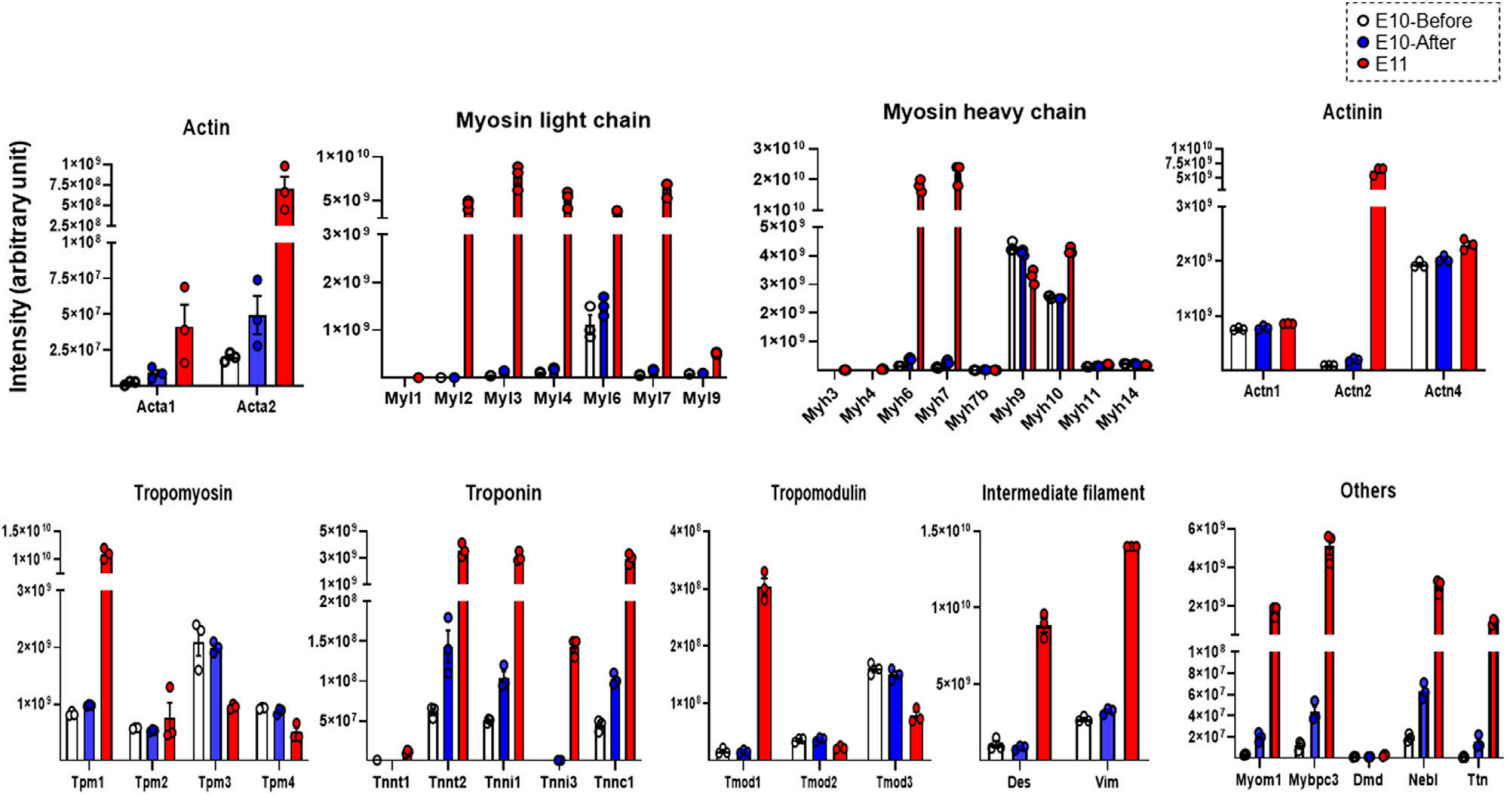
Expression levels of representative constituent proteins of myofibrils and sarcomeres assessed by DIA-MS in the heart primordium before and after heartbeat initiation and in the primitive heart tube. The expression levels of each protein obtained by DIA-MS proteome analysis for which both a peptide false discovery rate (FDR) < 1% and a protein FDR <1% are satisfied are represented for actin, myosin light chain, myosin heavy chain, actinin, tropomyosin, troponin, tropomodulin, intermediate filament and other components of myofilaments and sarcomeres. Values are expressed as intensity normalized by the median of all protein quantitation in each sample. Individual protein expression levels were simply represented as dot plots, means, and SEM in order to avoid multiple testing and data extraction errors in this small sample number (N = 3 each). Acta1, Actin, alpha skeletal muscle; Acta2, Actin, aortic smooth muscle; Myl1, Myosin light chain 1/3, skeletal muscle isoform; Myl2, Myosin regulatory light chain 2; ventricular/cardiac muscle isoform; Myl3, Myosin light chain 3; Myl4, Myosin light chain 4; Myl6, Myosin light polypeptide 6; Myl7, Myosin light chain 7; Myl9, Myosin regulatory light polypeptide 9; Myh3, Myosin-3; Myh4, Myosin-4; Myh6, Myosin-6; Myh7, Myosin-7; Myh7b, Myosin heavy chain 7B; Myh9, Myosin-9; Myh10, Myosin-10; Myh11, Myosin-11; Myh14, Myosin heavy chain 14; Actn1, Alpha-actinin-1; Actn2, Alpha-actinin-2; Actn4, Alpha-actinin-4; Tpm1, Tropomyosin alpha-1 chain; Tpm2, Tropomyosin beta chain; Tpm3, Tropomyosin alpha-3 chain; Tpm4, Tropomyosin alpha-4 chain; Tnnt1, Troponin T, slow skeletal muscle; Tnnt2, Troponin T, cardiac muscle; Tnni1, Troponin I, slow skeletal muscle; Tnni3, Troponin I, cardiac muscle; Tnnc1, Cardiac troponin C; Tmod1, Tropomodulin-1; Tmod2, Tropomodulin-2; Tmod3, Tropomodulin-3; Des, Desmin; Vim, Vimentin; Myom1, Myomesin 1; Mybpc3, Myosin-binding protein C, cardiac-type; Dmd, Dystrophin; Neb, Nebulette; Ttn, Cardiac titin fetal N2BA PEVK isoform.

Finally, we evaluated expression levels of individual constituent proteins of myofibrils and sarcomeres based on the listed molecules in the striated-muscle contraction pathway of Wikipathways (http://www.wikipathways.org) from the detected proteins assessed by DIA-MS proteome analysis. As shown in Figure 3, expression levels of individual proteins that are constituents of actin, myosin light chain, myosin heavy chain, actinin, tropomyosin, troponin, tropomodulin, intermediate filaments and other related molecules were evaluated. The expression levels of many constituent proteins of myofibrils and sarcomeres were considerably increased with development, being consistent with the findings of TEM observation that bundles of myofilaments were present in the heart primordium after heartbeat initiation and that sarcomeres were present in the primitive heart tube.

## Discussion

The initiation of heartbeat is an essential physiological event in embryonic development; however, how myofibrillogenesis is involved in the initial heartbeat has not been clarified. In the present study, we demonstrated that bundles of myofilaments, which were not observed in the heart primordium before heartbeat initiation, were present in the heart primordium just after heartbeat initiation and that myofibrils with sarcomere structures, though they were incomplete, were present in the state of the primitive heart tube (Figure 1). Since most previous studies using genetically modified animals focused only on the effects of genes on the development of fetal circulation, myocardial morphology, and embryonic lethality, it has remained unclear how the expression of proteins that are associated with myofibrillogenesis is involved in the initiation of heartbeat. Interestingly, skeletal muscle type actin (Acta1) and smooth muscle type actin (Acta2), but not cardiac muscle type actin (Actc1), were identified in the heart primordium even after heartbeat initiation (Figure 3). Indeed, smooth muscle type of actin has been reported to be expressed in the early embryonic heart (Woodcock-Mitchell et al., 1998). On the other hand, the expression of cardiac types of myosin light chain (Myl3, Myl7) and myosin heavy chain (Myh6, Myh7) was significantly upregulated in the heart primordium after heartbeat initiation compared with that before heartbeat initiation (Figure 2A). These findings indicate the possibility that expression of cardiac type of myosin is responsible for the formation of bundles of myofilaments in the heart primordium after heartbeat initiation and that the initial heartbeat is induced by the combination of smooth muscle type of actin and cardiac type of myosin. It should be noted that a sarcomere structure was not identified in the heart primordium after heartbeat initiation (Figure 1). Considering the theory that mechanical force is an important factor for myofibrillogenesis and sarcomere assembly (Lemke and Schnorrer. 2017), it is plausible that sarcomeres are not required at the onset of heartbeat but are formed in parallel with embryonic heart development, which requires tension to circulate the blood. In addition, our proteome analysis revealed that titin, the third most abundant protein in muscle next to actin and myosin, started to be expressed in the heart primordium after heartbeat initiation and that its expression level was dramatically increased in the primitive heart tube (Figure 3), supporting the notion that the expression of titin leads to the formation of sarcomeres (Maruyama. 1997). Since our study showed that the expression of many other important myofibrillogenesis-related proteins besides actin, myosin, and titin was also significantly upregulated in the heart primordium after heartbeat initiation (Figure 2A), further studies are needed to examine the distinct roles of these proteins in the initial heartbeat using genetically engineered animals.

It should be noted that bundles of myofilaments were identified in the present study only in a few cells of the heart primordium after heartbeat initiation despite upregulation of the expression of many proteins that are associated with myofibrils (Figures 1, 2A). We hypothesize that the findings can be explained by two possibilities. The first possibility is that the heartbeat in the whole heart primordium is provided only by some myofilament-expressing cells and that the other cells are just pulled by those cells, making the whole area appear to contract. The second possibility is that increased expression of myofilaments without forming bundles of myofilaments such as smooth muscle (Gunst and Zhang. 2008) can induce beating in the heart primordium because the heart primordium does not form a cylinder and stronger force is not needed for it to contract. Nevertheless, given previous reports showing that initial heartbeat (Kobayashi et al., 2011) or cardiomyoblast differentiation (Tyser et al., 2021) begins in the center of the heart primordium, it is reasonable that cells in the center of the heart primordium, even if only a few, play an important role in the initial heartbeat.

The results of our study also may provide a key for explaining the mechanisms of the findings in previous studies that calcium transients can precede regular heartbeat in rodent animals (Kobayashi et al., 2011; Tyser et al., 2016). That is, we hypothesize that the calcium transient, either transiently or continuously, already occurs prior to the development of a contractile apparatus that is necessary for heartbeat initiation and that the initial heartbeat is induced by cells in which the contractile apparatus is just about to be expressed. Indeed, previous studies showed that the calcium transient plays an important role in myofibrillogenesis in *xenopus* (Ferrari et al., 1996; Campbell et al., 2006). The preceding increase in intracellular calcium *via* the calcium transient may be a trigger for myofibrillogenesis and heartbeat initiation in species other than *xenopus*. In contrast, it remains to be determined if heartbeat itself promotes cardiac plasticity. Further studies are needed to determine the effect of the presence or absence of beating on subsequent development of the embryonic heart.

There are several limitations in this study. The cell specificity and the spatial resolution of protein profiling were not clarified because the proteome analysis was performed on isolated whole heart primordia or primitive heart tubes. To solve this issue, further studies using *in situ* single-cell proteome analysis are needed. In addition, we acknowledge that this brief report is descriptive without the results of functional assays. Especially, the force in the heart primordium after heartbeat initiation was not measured because of technological limitations. We consider that physiological studies focusing on excitation-contraction coupling at the timing of initial heartbeat are needed in the future.

In summary, we assessed the association between ultrastructural characteristics of myofibrils and sarcomeres and results of proteome analysis in the rat heart primordium before and after heartbeat initiation and we extended the investigation to the primitive heart tube. Our results indicate that initial heartbeat can be induced by upregulation of the expression of proteins that are associated with myofibrillogenesis, whereas complete sarcomere formation is, at least, not required for the initial heartbeat.

## Data Availability

The datasets of proteomic analysis presented in this study have been deposited in the ProteomeXchange Consortium via the jPOST partner repository (http://jpostdb.org) with the dataset identifier PXD032987 for ProteomeXchange and JPST001543for jPOST. Results are also found in online Supplementary Material.
